# Crystal structure of (1*S*,3*R*,8*R*,9*R*)-2,2-di­chloro-3,7,7-tri­methyl-10-methylenetri­cyclo­[6.4.0.0^1,3^]dodecan-9-ol

**DOI:** 10.1107/S2056989016011166

**Published:** 2016-07-22

**Authors:** Ahmed Benzalim, Aziz Auhmani, Abdoullah Bimoussa, My Youssef Ait Itto, Jean-Claude Daran, Abdelwahed Auhmani

**Affiliations:** a Laboratoire de Physico-Chimie Moléculaire et Synthèse Organique, Département de Chimie, Faculté des Sciences, Semlalia BP 2390, Marrakech 40001, Morocco; b Laboratoire de Chimie de Coordination, CNRS UPR8241, 205 route de Narbonne, 31077 Toulouse Cedex 04, France

**Keywords:** crystal structure, absolute configuration, sesquiterpenes, asymmetric synthesis, natural products, crystal structure

## Abstract

The title compound, C_16_H_24_Cl_2_O, was synthesized by treating (1*S*,3*R*,8*S*,9*R*,10*S*)-2,2-di­chloro-3,7,7,10-tetra­methyl-9,10-ep­oxy­tri­cyclo­[6.4.0.0^1,3^]dodecane with a concentrated solution of hydro­bromic acid. It is built up from three fused rings: a cyclo­heptane ring, a cyclo­hexyl ring bearing alkene and hy­droxy substituents, and a cyclo­propane ring bearing two chlorine atoms. The asymmetric unit contains two mol­ecules linked by an O—H⋯O hydrogen bond. In the crystal, further O—H⋯O hydrogen bonds build up an *R*_4_^4^(8) cyclic tetra­mer. One of the mol­ecules presents disorder that affects the seven-membered ring. In both mol­ecules, the six-membered rings display a chair conformation, whereas the seven-membered rings display conformations inter­mediate between boat and twist-boat for the non-disordered mol­ecule and either a chair or boat and twist-boat for the disordered mol­ecule owing to the disorder. The absolute configuration for both mol­ecules is 1*S*,3*R*,8*R*,9*R* and was deduced from the chemical pathway and further confirmed by the X-ray structural analysis.

## Abstract 2

The title compound was synthesized by treating (1*S*,3*R*,8*S*,9*R*,10*S*)-2,2-di­chloro-3,7,7,10-tetra­methyl-9,10-ep­oxy­tri­cyclo­[6.4.0.0^1,3^]dodecane with a concentrated solution of hydro­bromic acid. It is built up from three fused rings: a cyclo­heptane ring, a cyclo­hexyl ring bearing alkene and hy­droxy substituents, and a cyclo­propane ring bearing two chlorine atoms.

## Chemical context   

The main constituent (50%) of the essential oil of the Atlas cedar (*Cedrus atlantica*) is a bicyclic hydro­carbon sesquiterpene called β-himachalene (Plattier & Teisseire, 1974 ▸; Joseph & Dev, 1968 ▸). The reactivity of this sesquiterpene and its derivatives has been studied extensively (Auhmani *et al.*, 2002 ▸; El Jamili *et al.*, 2002 ▸; Dakir *et al.*, 2004 ▸). Optically active allylic alcohols are very inter­esting building inter­mediates that have been widely used in organic transformations (Paresh & Sujit, 2012 ▸; Arfaoui *et al.*, 2010 ▸). Several potent biologically active compounds contain this allylic alcohol functionality (Chung *et al.*, 2007 ▸; Servi *et al.*, 2000 ▸). In order to prepare new optically active allylic alcohols using this sesquiterpene, we prepared the title compound (1*S*,3*R*,8*R*,9*R*)-2,2-di­chloro-10-methyl­ene-3,7,7-tri­methyl­tri­cyclo­[6.4.0.0^1,3^]dodecan-9-ol by treating (1*S*,3*R*,8*S*,9*R*,10*S*)-2,2-di­chloro-3,7,7,10-tetra­methyl-9,10-ep­oxy­tri­cyclo­[6.4.0.0^1,3^]dodecane with a concentrated solution of hydro­bromic acid.

## Structural commentary   

**Figure 1:**
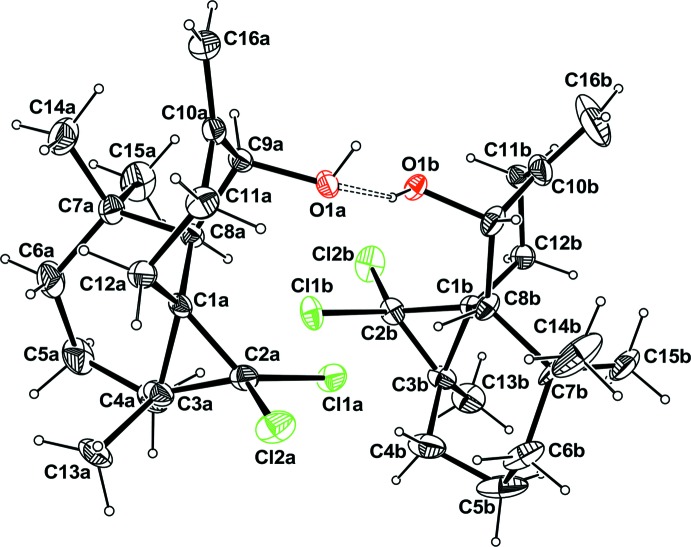
The mol­ecular view of the title compound with the atom-labeling scheme. For clarity, only one component of the disorder is represented. Ellipsoids are drawn at the 30% probability level. H atoms are represented as small circles of arbitrary radius. The hydrogen bond is represented as dashed line.

There are two mol­ecules *A* and *B* within the asymmetric unit, which are built up from three fused rings, a seven-membered heptane ring, a six-membered cyclo­hexyl ring bearing an hydroxyl and alkene groups and a three-membered propane ring bearing two Cl atoms (Fig. 1 ▸). 
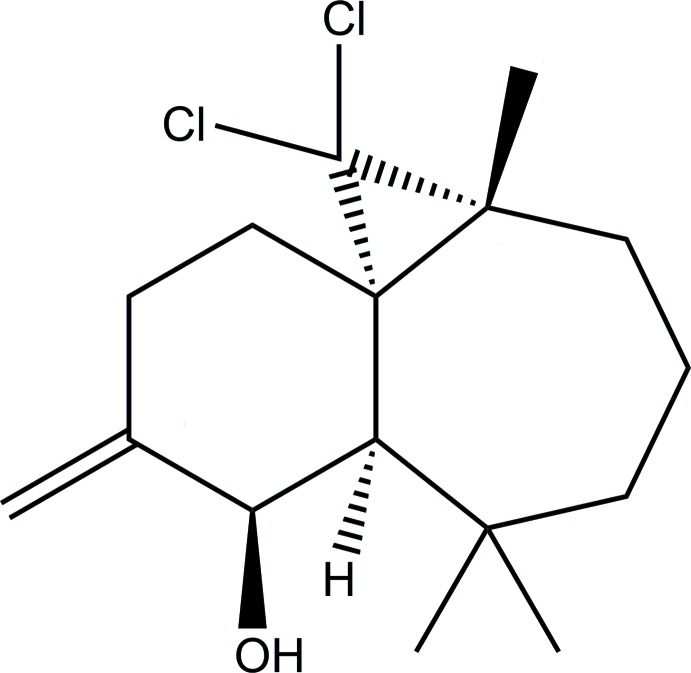


**Figure 2:**
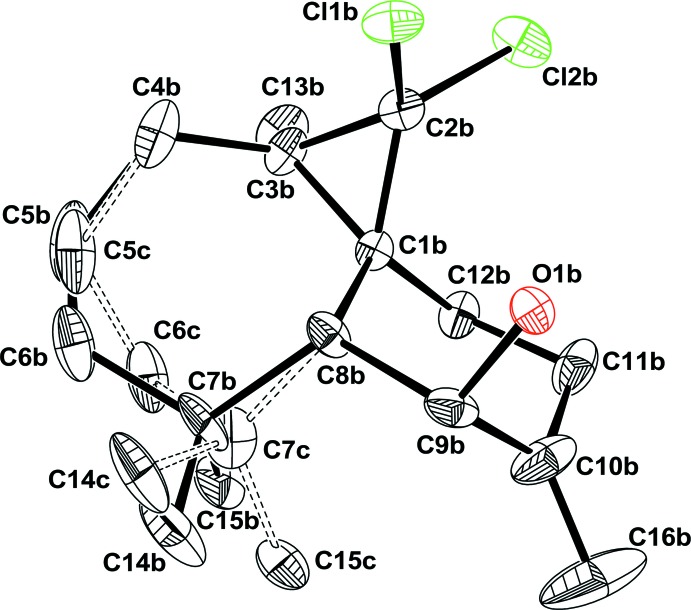
View showing the disorder in mol­ecule *B*. Bonds in the minor disorder component are shown as dashed lines.

In mol­ecule *B*, there is disorder affecting the location of the C5*B*, C6*B*, and C7*B* atoms, which are split over two positions C5*C*, C6*C*, and C7*C* (Fig. 2 ▸), resulting in disorder of the two methyl atoms attached to C7*B* and C7*C*, and also disorder for the two H atoms attached to C5*B* and C5*C*. The disordered sites have occupancy factor in the ratio 0.502 (8):0.498 (8). In both mol­ecules, the six-membered ring displays a chair conformation with puckering parameters θ = 169.3° and φ_2_ = 119.6° for mol­ecule *A* and θ = 172.1° and φ_2_ = 110.0° for mol­ecule *B*. The seven-membered cyclo­heptane ring in mol­ecule *A* displays a conformation inter­mediate between boat and twist boat with puckering parameters *q*_2_ = 1.138 (4) Å and *q*_3_ = 0.037 (5) Å (Boessenkool & Boeyens, 1980 ▸). As a result of the disorder observed in mol­ecule *B* within the seven-membered ring, the conformation of this ring is inter­mediate between chair [*q*_2_ = 0.434 (6), *q*_3_ = 0.739 (6) Å] or boat and twist-boat [*q*_2_ = 1.173 (5), *q*_3_ = 0.020 (4) Å] (Boessenkool & Boeyens, 1980 ▸), depending on the position of the C6*B*(*C*) atom. The disorder does not affect the absolute configuration of the two mol­ecules (1*AS*,3*AR*,8*AR*,9*AR*) and (1*BS*,3*BR*,8*BR*,9*BR*).

## Supra­molecular features   

**Figure 3:**
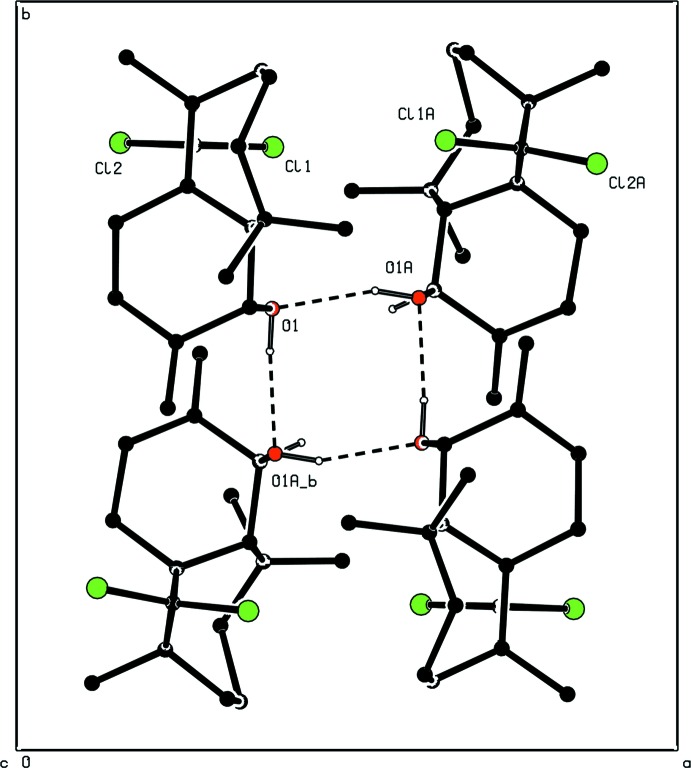
Partial packing diagram (*PLUTON;* Spek, 2009 ▸) showing the formation of the 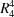
(8) tetra­mer. H atoms not involved in hydrogen bonding have been removed for the sake of clarity.

The two independent mol­ecules are connected by O—H⋯O hydrogen bonds (Table 1 ▸), building a pseudo-dimer. Pairs of such dimers are connected by O—H⋯O hydrogen bonds, building an *R* ^4^4(8) cyclic tetra­mer (Fig. 3 ▸). There are also weak C—H⋯Cl intra­molecular inter­actions (Table 1 ▸).

## Database survey   

A search of the Cambridge Structural Database (CSD, Version 5.37, update November 2015; Groom *et al.*, 2016 ▸) using fused cyclo­hexyl, cyclo­heptane and cyclo­propane rings system as the main skeleton, revealed the presence of 32 structures. Among these, only one, C_16_H_22_Br_2_Cl_2_ (Auhmani *et al.*, 2002 ▸), contains a cyclo­hexyl ring substituted by a =CH_2_ group but, to the best of our knowledge, there are no reported structures that have a cyclo­hexyl group substituted by a hydroxyl at C9*A* (C9*B*).

## Synthesis and crystallization   

To a 100 mL flask was added (1 g, 3.29 mmol) of (1*S*,3*R*,8*S*,9*R*,10*S*)-2,2- di­chloro-3,7,7,10-tetra­methyl-9,10-ep­oxy­tri­cyclo­[6.4.0.0^1,3^]dodecane in 25 mL of di­chloro­methane. The mixture was cooled to 273.15 K in an ice bath prior to dropwise addition of 8 mL of concentrated hydro­bromic acid. The mixture was stirred for 30 min. TLC control showed that the reaction was complete. The reaction mixture was extracted with di­chloro­methane (3 × 30mL) and the organic layer was washed first with water and then with a saturated solution of NaHCO_3_, dried over anhydrous Na_2_SO_4_ and concentrated under reduced pressure. The crude product was purified by chromatography on silica gel (230–400 mesh) with hexa­ne/ethyl acetate (97:3) as eluent to give the title compound in 64% yield. X-ray quality crystals were obtained by slow evaporation from a petroleum ether solution.

## Refinement   

Crystal data, data collection and structure refinement details are summarized in Table 2 ▸. All H atoms were initially located in a difference Fourier map but they were placed in geometrically idealized positions and constrained to ride on their parent atoms with C—H distances = 1.0 Å (Cmethine), 0.98 Å (Cmeth­yl), 0.99 Å (Cmethyl­ene) and 0.84 Å (hydrox­yl), with *U*_iso_(H) = 1.2*U*_eq_(C_methine_, C_methyl­ene_) or 1.5*U*_eq_(C_meth­yl_, O_hydrox­yl_).

## Supplementary Material

Crystal structure: contains datablock(s) I, global. DOI: 10.1107/S2056989016011166/pk2583sup1.cif

Structure factors: contains datablock(s) I. DOI: 10.1107/S2056989016011166/pk2583Isup2.hkl

Supporting information file. DOI: 10.1107/S2056989016011166/pk2583Isup3.cml

CCDC reference: 1491732

Additional supporting information: crystallographic information; 3D view; checkCIF report

## Figures and Tables

**Table 1:** Hydrogen-bond geometry (Å, °)

*D*—H⋯*A*	*D*—H	H⋯*A*	*D*⋯*A*	*D*—H⋯*A*
O1*A*—H1*A*⋯O1*B* ^i^	0.82	1.98	2.791 (4)	169
C8*A*—H8*A*⋯Cl1*A*	0.98	2.68	3.228 (4)	116
O1*B*—H1*B*⋯O1*A*	0.82	2.04	2.835 (4)	162
C8*B*—H8*B*⋯Cl1*A*	0.98	2.78	3.691 (4)	156
C8*B*—H8*B*⋯Cl1*B*	0.98	2.63	3.238 (4)	120
O1*A*—H1*A*⋯O1*B* ^i^	0.82	1.98	2.791 (4)	169
C8*A*—H8*A*⋯Cl1*A*	0.98	2.68	3.228 (4)	116
O1*B*—H1*B*⋯O1*A*	0.82	2.04	2.835 (4)	162
C8*B*—H8*B*⋯Cl1*A*	0.98	2.78	3.691 (4)	156
C8*B*—H8*B*⋯Cl1*B*	0.98	2.63	3.238 (4)	120

Symmetry code: (i) 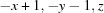.

**Table 2:** Experimental details

Crystal data	
Chemical formula	C_16_H_24_Cl_2_O
*M*_r_	303.25
Crystal system, space group	Orthorhombic, *P*2_1_2_1_2
Temperature (K)	180
*a*, *b*, *c* (Å)	12.3075 (4), 13.9332 (7), 18.6716 (9)
*V* (Å^3^)	3201.9 (2)
*Z*	8
Radiation type	Mo *K*α
μ (mm^−1^)	0.40
Crystal size (mm)	0.43 × 0.31 × 0.25

Data collection	
Diffractometer	Agilent Xcalibur Eos Gemini ultra
Absorption correction	Multi-scan (*CrysAlis PRO*; Agilent, 2014 ▸)
*T*_min_, *T*_max_	0.907, 1.000
No. of measured, independent and observed [*I* > 2σ(*I*)] reflections	20074, 7060, 6084
*R*_int_	0.033
(sin θ/λ)_max_ (Å^−1^)	0.641

Refinement	
*R*[*F* ^2^ > 2σ(*F* ^2^)], *wR*(*F* ^2^), *S*	0.047, 0.121, 1.06
No. of reflections	7060
No. of parameters	393
No. of restraints	22
H-atom treatment	H-atom parameters constrained
Δρ_max_, Δρ_min_ (e Å^−3^)	0.52, −0.36
Absolute structure	Flack *x* determined using 2376 quotients [(*I* ^+^)−(*I* ^−^)]/[(*I* ^+^)+(*I* ^−^)] (Parsons *et al.*, 2013 ▸)
Absolute structure parameter	0.03 (3)

Computer programs: *CrysAlis PRO* (Agilent, 2014 ▸), *SHELXT2013*(Sheldrick, 2015*a* ▸), *SHELXL2013* (Sheldrick, 2015*b* ▸), *ORTEPIII* (Burnett & Johnson, 1996 ▸), *ORTEP-3 for Windows* (Farrugia, 2012 ▸), *Mercury* (Macrae *et al.*, 2008 ▸) and *PLATON* (Spek, 2009 ▸).

## supplementary crystallographic information

### Crystal data

**Table:**

C_16_H_24_Cl_2_O	*D*_x_ = 1.258 Mg m^−^^3^
*M**_r_* = 303.25	Mo *K*α radiation, λ = 0.71073 Å
Orthorhombic, *P*2_1_2_1_2	Cell parameters from 5974 reflections
*a* = 12.3075 (4) Å	θ = 3.5–30.6°
*b* = 13.9332 (7) Å	µ = 0.40 mm^−^^1^
*c* = 18.6716 (9) Å	*T* = 180 K
*V* = 3201.9 (2) Å^3^	Box, colourless
*Z* = 8	0.43 × 0.31 × 0.25 mm
*F*(000) = 1296	

### Data collection

**Table:**

Agilent Xcalibur Eos Gemini ultra diffractometer	7060 independent reflections
Radiation source: Enhance (Mo) X-ray Source	6084 reflections with *I* > 2σ(*I*)
Graphite monochromator	*R*_int_ = 0.033
Detector resolution: 16.1978 pixels mm^-1^	θ_max_ = 27.1°, θ_min_ = 2.9°
ω scans	*h* = −15→15
Absorption correction: multi-scan (CrysAlis PRO; Agilent, 2014)	*k* = −17→17
*T*_min_ = 0.907, *T*_max_ = 1.000	*l* = −23→23
20074 measured reflections	

### Refinement

**Table:**

Refinement on *F*^2^	Secondary atom site location: difference Fourier map
Least-squares matrix: full	Hydrogen site location: inferred from neighbouring sites
*R*[*F*^2^ > 2σ(*F*^2^)] = 0.047	H-atom parameters constrained
*wR*(*F*^2^) = 0.121	*w* = 1/[σ^2^(*F*_o_^2^) + (0.0522*P*)^2^ + 1.8807*P*] where *P* = (*F*_o_^2^ + 2*F*_c_^2^)/3
*S* = 1.06	(Δ/σ)_max_ < 0.001
7060 reflections	Δρ_max_ = 0.52 e Å^−^^3^
393 parameters	Δρ_min_ = −0.36 e Å^−^^3^
22 restraints	Absolute structure: Flack *x* determined using 2376 quotients [(*I*^+^)-(*I*^-^)]/[(*I*^+^)+(*I*^-^)] (Parsons *et al.*, 2013)
Primary atom site location: structure-invariant direct methods	Absolute structure parameter: 0.03 (3)

### Special details

**Table:**

Experimental. Empirical absorption correction using spherical harmonics, implemented in SCALE3 ABSPACK scaling algorithm, CrysAlisPro (Agilent Technologies, 2014)
Geometry. All esds (except the esd in the dihedral angle between two l.s. planes) are estimated using the full covariance matrix. The cell esds are taken into account individually in the estimation of esds in distances, angles and torsion angles; correlations between esds in cell parameters are only used when they are defined by crystal symmetry. An approximate (isotropic) treatment of cell esds is used for estimating esds involving l.s. planes.

### Fractional atomic coordinates and isotropic or equivalent isotropic displacement parameters (Å^2^)

**Table:**

	*x*	*y*	*z*	*U*_iso_*/*U*_eq_	Occ. (<1)
Cl1A	0.38798 (7)	−0.19453 (7)	−0.28372 (5)	0.0380 (2)	
Cl2A	0.15680 (8)	−0.18866 (9)	−0.29099 (6)	0.0479 (3)	
O1A	0.3867 (2)	−0.41044 (17)	−0.22969 (13)	0.0298 (5)	
H1A	0.3817	−0.4654	−0.2451	0.045*	
C1A	0.2588 (3)	−0.2464 (2)	−0.16119 (19)	0.0257 (7)	
C2A	0.2689 (3)	−0.1917 (3)	−0.2314 (2)	0.0316 (8)	
C3A	0.2659 (3)	−0.1359 (3)	−0.1627 (2)	0.0362 (9)	
C4A	0.3710 (4)	−0.0925 (3)	−0.1351 (2)	0.0461 (11)	
H4A1	0.3738	−0.0252	−0.1485	0.055*	
H4A2	0.4320	−0.1246	−0.1577	0.055*	
C5A	0.3822 (5)	−0.1010 (3)	−0.0532 (3)	0.0570 (13)	
H5A1	0.4586	−0.0970	−0.0408	0.068*	
H5A2	0.3459	−0.0467	−0.0312	0.068*	
C6A	0.3356 (5)	−0.1933 (3)	−0.0211 (2)	0.0573 (13)	
H6A1	0.2575	−0.1914	−0.0276	0.069*	
H6A2	0.3491	−0.1915	0.0301	0.069*	
C7A	0.3755 (4)	−0.2906 (3)	−0.0487 (2)	0.0372 (9)	
C8A	0.3567 (3)	−0.3021 (2)	−0.13200 (18)	0.0256 (7)	
H8A	0.4208	−0.2742	−0.1551	0.031*	
C9A	0.3528 (3)	−0.4083 (2)	−0.15632 (18)	0.0272 (7)	
H9A	0.4042	−0.4456	−0.1275	0.033*	
C10A	0.2414 (3)	−0.4547 (3)	−0.1516 (2)	0.0324 (9)	
C11A	0.1495 (3)	−0.3959 (3)	−0.1800 (2)	0.0370 (9)	
H11A	0.0812	−0.4273	−0.1690	0.044*	
H11B	0.1556	−0.3911	−0.2317	0.044*	
C12A	0.1503 (3)	−0.2955 (3)	−0.1473 (2)	0.0322 (8)	
H12A	0.0921	−0.2575	−0.1681	0.039*	
H12B	0.1378	−0.2999	−0.0961	0.039*	
C13A	0.1662 (4)	−0.0747 (3)	−0.1461 (3)	0.0516 (12)	
H13A	0.1720	−0.0143	−0.1706	0.077*	
H13B	0.1619	−0.0637	−0.0954	0.077*	
H13C	0.1019	−0.1075	−0.1619	0.077*	
C14A	0.3170 (5)	−0.3675 (4)	−0.0029 (2)	0.0568 (13)	
H14A	0.3322	−0.3566	0.0468	0.085*	
H14B	0.3425	−0.4301	−0.0163	0.085*	
H14C	0.2401	−0.3636	−0.0109	0.085*	
C15A	0.4969 (4)	−0.3035 (4)	−0.0350 (3)	0.0612 (14)	
H15A	0.5365	−0.2538	−0.0592	0.092*	
H15B	0.5197	−0.3650	−0.0528	0.092*	
H15C	0.5109	−0.3000	0.0155	0.092*	
C16A	0.2287 (4)	−0.5428 (3)	−0.1266 (2)	0.0436 (10)	
H16A	0.1601	−0.5709	−0.1262	0.052*	
H16B	0.2883	−0.5768	−0.1094	0.052*	
Cl1B	0.64962 (7)	−0.18578 (8)	−0.20137 (5)	0.0415 (2)	
Cl2B	0.87789 (8)	−0.21670 (9)	−0.20248 (6)	0.0518 (3)	
O1B	0.6090 (2)	−0.39598 (19)	−0.26805 (12)	0.0302 (5)	
H1B	0.5438	−0.3880	−0.2610	0.045*	
C1B	0.7575 (3)	−0.2429 (3)	−0.33159 (19)	0.0262 (8)	
C2B	0.7636 (3)	−0.1965 (3)	−0.2577 (2)	0.0308 (8)	
C3B	0.7751 (3)	−0.1342 (3)	−0.3232 (2)	0.0356 (9)	
C4B	0.6812 (4)	−0.0682 (3)	−0.3428 (3)	0.0572 (14)	
H4B1	0.6939	−0.0050	−0.3226	0.069*	
H4B2	0.6144	−0.0932	−0.3225	0.069*	
C5B	0.669 (2)	−0.0598 (13)	−0.4252 (9)	0.075 (10)	0.498 (8)
H5B1	0.6455	0.0041	−0.4387	0.090*	0.498 (8)
H5B2	0.7371	−0.0739	−0.4491	0.090*	0.498 (8)
C6B	0.5817 (8)	−0.1352 (7)	−0.4443 (6)	0.055 (3)	0.498 (8)
H6B1	0.5203	−0.1263	−0.4125	0.067*	0.498 (8)
H6B2	0.5566	−0.1226	−0.4927	0.067*	0.498 (8)
C14B	0.517 (2)	−0.2953 (16)	−0.4674 (17)	0.084 (10)	0.498 (8)
H14D	0.4980	−0.2721	−0.5141	0.126*	0.498 (8)
H14E	0.4574	−0.2851	−0.4351	0.126*	0.498 (8)
H14F	0.5331	−0.3626	−0.4700	0.126*	0.498 (8)
C15B	0.7119 (9)	−0.2558 (8)	−0.4924 (5)	0.054 (3)	0.498 (8)
H15D	0.7343	−0.3218	−0.4912	0.082*	0.498 (8)
H15E	0.7718	−0.2155	−0.4789	0.082*	0.498 (8)
H15F	0.6887	−0.2395	−0.5400	0.082*	0.498 (8)
C7B	0.6177 (18)	−0.2408 (11)	−0.4400 (15)	0.043 (3)	0.498 (8)
C5C	0.656 (2)	−0.0716 (12)	−0.4234 (7)	0.064 (8)	0.502 (8)
H5C1	0.6916	−0.0176	−0.4462	0.077*	0.502 (8)
H5C2	0.5781	−0.0626	−0.4295	0.077*	0.502 (8)
C6C	0.6896 (8)	−0.1660 (6)	−0.4647 (5)	0.059 (3)	0.502 (8)
H6C1	0.6757	−0.1556	−0.5152	0.071*	0.502 (8)
H6C2	0.7675	−0.1738	−0.4592	0.071*	0.502 (8)
C14C	0.5122 (19)	−0.2525 (16)	−0.4543 (15)	0.073 (7)	0.502 (8)
H14G	0.4976	−0.2365	−0.5034	0.110*	0.502 (8)
H14H	0.4846	−0.2028	−0.4238	0.110*	0.502 (8)
H14I	0.4775	−0.3122	−0.4428	0.110*	0.502 (8)
C15C	0.6759 (9)	−0.3422 (7)	−0.4933 (4)	0.054 (3)	0.502 (8)
H15G	0.6322	−0.3986	−0.4867	0.081*	0.502 (8)
H15H	0.7503	−0.3568	−0.4823	0.081*	0.502 (8)
H15I	0.6706	−0.3212	−0.5422	0.081*	0.502 (8)
C7C	0.6353 (18)	−0.2623 (11)	−0.4434 (14)	0.043 (3)	0.502 (8)
C8B	0.6477 (3)	−0.2777 (3)	−0.36065 (19)	0.0314 (8)	
H8B	0.5944	−0.2454	−0.3300	0.038*	
C9B	0.6317 (4)	−0.3854 (3)	−0.3432 (2)	0.0401 (10)	
H9B	0.5703	−0.4103	−0.3710	0.048*	
C10B	0.7308 (5)	−0.4468 (3)	−0.3572 (3)	0.0603 (15)	
C11B	0.8344 (4)	−0.4091 (3)	−0.3272 (3)	0.0531 (13)	
H11C	0.8943	−0.4494	−0.3426	0.064*	
H11D	0.8314	−0.4110	−0.2753	0.064*	
C12B	0.8540 (3)	−0.3069 (3)	−0.3517 (2)	0.0365 (9)	
H12C	0.8643	−0.3058	−0.4032	0.044*	
H12D	0.9196	−0.2823	−0.3294	0.044*	
C13B	0.8857 (4)	−0.0895 (3)	−0.3402 (3)	0.0516 (12)	
H13D	0.8978	−0.0353	−0.3095	0.077*	
H13E	0.8869	−0.0689	−0.3893	0.077*	
H13F	0.9419	−0.1362	−0.3326	0.077*	
C16B	0.7226 (8)	−0.5295 (5)	−0.3905 (4)	0.122 (4)	
H16C	0.7837	−0.5679	−0.3964	0.147*	
H16D	0.6558	−0.5495	−0.4083	0.147*	

### Atomic displacement parameters (Å^2^)

**Table:**

	*U*^11^	*U*^22^	*U*^33^	*U*^12^	*U*^13^	*U*^23^
Cl1A	0.0268 (4)	0.0437 (5)	0.0436 (5)	0.0046 (4)	0.0118 (4)	0.0125 (4)
Cl2A	0.0306 (5)	0.0585 (6)	0.0545 (6)	0.0073 (5)	−0.0058 (4)	0.0172 (6)
O1A	0.0319 (12)	0.0283 (12)	0.0293 (12)	0.0013 (11)	0.0054 (11)	−0.0061 (10)
C1A	0.0208 (17)	0.0238 (17)	0.0326 (18)	0.0037 (13)	0.0079 (14)	−0.0010 (14)
C2A	0.0213 (15)	0.0320 (18)	0.041 (2)	0.0052 (17)	0.0068 (14)	0.0065 (17)
C3A	0.0295 (19)	0.0258 (18)	0.053 (2)	0.0023 (16)	0.0125 (18)	0.0034 (18)
C4A	0.048 (3)	0.0273 (18)	0.063 (3)	−0.0091 (19)	0.012 (2)	−0.0074 (19)
C5A	0.072 (3)	0.040 (2)	0.059 (3)	−0.016 (2)	0.006 (3)	−0.024 (2)
C6A	0.082 (4)	0.049 (3)	0.041 (2)	−0.004 (3)	0.008 (2)	−0.020 (2)
C7A	0.044 (2)	0.038 (2)	0.0295 (18)	−0.0011 (17)	−0.0010 (17)	−0.0053 (15)
C8A	0.0252 (16)	0.0249 (16)	0.0265 (16)	0.0032 (14)	−0.0008 (13)	−0.0016 (14)
C9A	0.0296 (17)	0.0242 (16)	0.0278 (17)	0.0053 (14)	−0.0014 (14)	−0.0016 (14)
C10A	0.041 (2)	0.0277 (18)	0.0285 (19)	−0.0029 (16)	0.0082 (16)	−0.0058 (15)
C11A	0.0293 (18)	0.037 (2)	0.045 (2)	−0.0118 (17)	0.0042 (16)	0.0015 (17)
C12A	0.0247 (17)	0.0319 (19)	0.040 (2)	0.0009 (15)	0.0075 (15)	0.0050 (15)
C13A	0.051 (3)	0.030 (2)	0.074 (3)	0.016 (2)	0.022 (2)	0.001 (2)
C14A	0.079 (4)	0.060 (3)	0.031 (2)	−0.013 (3)	0.004 (2)	−0.003 (2)
C15A	0.058 (3)	0.082 (4)	0.044 (3)	0.000 (3)	−0.023 (2)	−0.007 (3)
C16A	0.055 (3)	0.032 (2)	0.044 (2)	−0.007 (2)	0.011 (2)	0.0018 (18)
Cl1B	0.0315 (4)	0.0501 (5)	0.0429 (5)	−0.0046 (4)	0.0115 (4)	−0.0152 (5)
Cl2B	0.0275 (5)	0.0792 (8)	0.0487 (6)	−0.0090 (5)	−0.0076 (4)	−0.0049 (5)
O1B	0.0291 (12)	0.0364 (13)	0.0251 (12)	−0.0029 (12)	0.0062 (10)	0.0055 (10)
C1B	0.0196 (17)	0.0272 (18)	0.0319 (18)	−0.0015 (13)	0.0059 (14)	0.0033 (15)
C2B	0.0160 (15)	0.0367 (19)	0.040 (2)	−0.0046 (16)	0.0020 (13)	−0.0041 (17)
C3B	0.0270 (18)	0.0262 (18)	0.054 (2)	−0.0038 (16)	0.0087 (18)	0.0028 (17)
C4B	0.045 (3)	0.039 (2)	0.087 (4)	0.012 (2)	0.016 (3)	0.018 (2)
C5B	0.055 (13)	0.045 (9)	0.12 (2)	−0.006 (8)	−0.003 (12)	0.050 (10)
C6B	0.038 (5)	0.070 (7)	0.059 (6)	−0.002 (5)	−0.003 (4)	0.039 (5)
C14B	0.079 (13)	0.114 (18)	0.058 (13)	−0.050 (14)	−0.046 (10)	0.044 (14)
C15B	0.076 (7)	0.058 (7)	0.029 (4)	−0.013 (5)	0.000 (4)	0.018 (4)
C7B	0.041 (6)	0.057 (6)	0.030 (3)	−0.007 (5)	−0.002 (4)	0.016 (5)
C5C	0.038 (8)	0.078 (14)	0.077 (13)	0.013 (9)	0.027 (8)	0.061 (10)
C6C	0.054 (6)	0.073 (8)	0.049 (6)	0.002 (5)	0.010 (4)	0.041 (5)
C14C	0.049 (8)	0.12 (2)	0.047 (11)	−0.022 (11)	−0.012 (7)	0.052 (13)
C15C	0.068 (7)	0.073 (7)	0.022 (4)	−0.002 (5)	0.003 (4)	0.005 (4)
C7C	0.041 (6)	0.057 (6)	0.030 (3)	−0.007 (5)	−0.002 (4)	0.016 (5)
C8B	0.0309 (18)	0.039 (2)	0.0241 (17)	−0.0113 (16)	0.0011 (15)	0.0072 (15)
C9B	0.056 (3)	0.041 (2)	0.0233 (18)	−0.023 (2)	0.0019 (18)	0.0003 (15)
C10B	0.097 (4)	0.033 (2)	0.051 (3)	−0.014 (3)	0.043 (3)	−0.010 (2)
C11B	0.066 (3)	0.036 (2)	0.057 (3)	0.019 (2)	0.029 (2)	0.006 (2)
C12B	0.0325 (19)	0.0370 (19)	0.040 (2)	0.0046 (18)	0.0146 (16)	0.0047 (17)
C13B	0.037 (2)	0.043 (2)	0.075 (3)	−0.017 (2)	0.012 (2)	0.004 (2)
C16B	0.160 (8)	0.072 (4)	0.134 (7)	−0.044 (5)	0.092 (6)	−0.065 (4)

### Geometric parameters (Å, º)

**Table:**

Cl1A—C2A	1.762 (3)	C2B—C3B	1.506 (6)
Cl2A—C2A	1.773 (4)	C3B—C4B	1.521 (6)
O1A—C9A	1.432 (4)	C3B—C13B	1.531 (5)
O1A—H1A	0.8200	C4B—C5C	1.538 (15)
C1A—C2A	1.521 (5)	C4B—C5B	1.550 (16)
C1A—C12A	1.523 (5)	C4B—H4B1	0.9700
C1A—C8A	1.533 (5)	C4B—H4B2	0.9700
C1A—C3A	1.542 (5)	C5B—C6B	1.54 (2)
C2A—C3A	1.500 (6)	C5B—H5B1	0.9700
C3A—C4A	1.519 (6)	C5B—H5B2	0.9700
C3A—C13A	1.527 (6)	C6B—C7B	1.539 (16)
C4A—C5A	1.541 (7)	C6B—H6B1	0.9700
C4A—H4A1	0.9700	C6B—H6B2	0.9700
C4A—H4A2	0.9700	C14B—C7B	1.540 (16)
C5A—C6A	1.530 (7)	C14B—H14D	0.9600
C5A—H5A1	0.9700	C14B—H14E	0.9600
C5A—H5A2	0.9700	C14B—H14F	0.9600
C6A—C7A	1.531 (6)	C15B—C7B	1.532 (17)
C6A—H6A1	0.9700	C15B—H15D	0.9600
C6A—H6A2	0.9700	C15B—H15E	0.9600
C7A—C15A	1.527 (6)	C15B—H15F	0.9600
C7A—C14A	1.548 (6)	C7B—C8B	1.61 (3)
C7A—C8A	1.582 (5)	C5C—C6C	1.581 (13)
C8A—C9A	1.548 (5)	C5C—H5C1	0.9700
C8A—H8A	0.9800	C5C—H5C2	0.9700
C9A—C10A	1.518 (5)	C6C—C7C	1.550 (16)
C9A—H9A	0.9800	C6C—H6C1	0.9700
C10A—C16A	1.323 (5)	C6C—H6C2	0.9700
C10A—C11A	1.494 (6)	C14C—C7C	1.535 (17)
C11A—C12A	1.527 (5)	C14C—H14G	0.9600
C11A—H11A	0.9700	C14C—H14H	0.9600
C11A—H11B	0.9700	C14C—H14I	0.9600
C12A—H12A	0.9700	C15C—C7C	1.537 (16)
C12A—H12B	0.9700	C15C—H15G	0.9600
C13A—H13A	0.9600	C15C—H15H	0.9600
C13A—H13B	0.9600	C15C—H15I	0.9600
C13A—H13C	0.9600	C7C—C8B	1.57 (3)
C14A—H14A	0.9600	C8B—C9B	1.548 (5)
C14A—H14B	0.9600	C8B—H8B	0.9800
C14A—H14C	0.9600	C9B—C10B	1.512 (7)
C15A—H15A	0.9600	C9B—H9B	0.9800
C15A—H15B	0.9600	C10B—C16B	1.313 (7)
C15A—H15C	0.9600	C10B—C11B	1.489 (8)
C16A—H16A	0.9300	C11B—C12B	1.515 (6)
C16A—H16B	0.9300	C11B—H11C	0.9700
Cl1B—C2B	1.760 (4)	C11B—H11D	0.9700
Cl2B—C2B	1.767 (4)	C12B—H12C	0.9700
O1B—C9B	1.439 (4)	C12B—H12D	0.9700
O1B—H1B	0.8200	C13B—H13D	0.9600
C1B—C2B	1.524 (5)	C13B—H13E	0.9600
C1B—C12B	1.532 (5)	C13B—H13F	0.9600
C1B—C8B	1.534 (5)	C16B—H16C	0.9300
C1B—C3B	1.538 (5)	C16B—H16D	0.9300
			
C9A—O1A—H1A	109.5	C4B—C3B—C1B	117.6 (4)
C2A—C1A—C12A	116.3 (3)	C13B—C3B—C1B	120.3 (4)
C2A—C1A—C8A	119.8 (3)	C3B—C4B—C5C	111.9 (7)
C12A—C1A—C8A	113.7 (3)	C3B—C4B—C5B	111.0 (9)
C2A—C1A—C3A	58.6 (3)	C3B—C4B—H4B1	109.4
C12A—C1A—C3A	120.0 (3)	C5B—C4B—H4B1	109.4
C8A—C1A—C3A	117.9 (3)	C3B—C4B—H4B2	109.4
C3A—C2A—C1A	61.4 (2)	C5B—C4B—H4B2	109.4
C3A—C2A—Cl1A	120.4 (3)	H4B1—C4B—H4B2	108.0
C1A—C2A—Cl1A	122.3 (3)	C6B—C5B—C4B	104.2 (14)
C3A—C2A—Cl2A	120.4 (3)	C6B—C5B—H5B1	110.9
C1A—C2A—Cl2A	119.3 (3)	C4B—C5B—H5B1	110.9
Cl1A—C2A—Cl2A	107.4 (2)	C6B—C5B—H5B2	110.9
C2A—C3A—C4A	118.4 (3)	C4B—C5B—H5B2	110.9
C2A—C3A—C13A	118.9 (4)	H5B1—C5B—H5B2	108.9
C4A—C3A—C13A	113.1 (4)	C7B—C6B—C5B	116.0 (13)
C2A—C3A—C1A	60.0 (2)	C7B—C6B—H6B1	108.3
C4A—C3A—C1A	116.1 (4)	C5B—C6B—H6B1	108.3
C13A—C3A—C1A	120.6 (4)	C7B—C6B—H6B2	108.3
C3A—C4A—C5A	112.4 (4)	C5B—C6B—H6B2	108.3
C3A—C4A—H4A1	109.1	H6B1—C6B—H6B2	107.4
C5A—C4A—H4A1	109.1	C7B—C14B—H14D	109.5
C3A—C4A—H4A2	109.1	C7B—C14B—H14E	109.5
C5A—C4A—H4A2	109.1	H14D—C14B—H14E	109.5
H4A1—C4A—H4A2	107.9	C7B—C14B—H14F	109.5
C6A—C5A—C4A	114.9 (4)	H14D—C14B—H14F	109.5
C6A—C5A—H5A1	108.5	H14E—C14B—H14F	109.5
C4A—C5A—H5A1	108.5	C7B—C15B—H15D	109.5
C6A—C5A—H5A2	108.5	C7B—C15B—H15E	109.5
C4A—C5A—H5A2	108.5	H15D—C15B—H15E	109.5
H5A1—C5A—H5A2	107.5	C7B—C15B—H15F	109.5
C5A—C6A—C7A	119.5 (4)	H15D—C15B—H15F	109.5
C5A—C6A—H6A1	107.4	H15E—C15B—H15F	109.5
C7A—C6A—H6A1	107.4	C15B—C7B—C6B	108.3 (13)
C5A—C6A—H6A2	107.4	C15B—C7B—C14B	109 (2)
C7A—C6A—H6A2	107.4	C6B—C7B—C14B	102.9 (15)
H6A1—C6A—H6A2	107.0	C15B—C7B—C8B	111.8 (15)
C15A—C7A—C6A	111.2 (4)	C6B—C7B—C8B	114.8 (14)
C15A—C7A—C14A	106.3 (4)	C14B—C7B—C8B	109.4 (16)
C6A—C7A—C14A	106.2 (4)	C4B—C5C—C6C	116.6 (11)
C15A—C7A—C8A	107.2 (3)	C4B—C5C—H5C1	108.1
C6A—C7A—C8A	112.0 (3)	C6C—C5C—H5C1	108.1
C14A—C7A—C8A	113.8 (3)	C4B—C5C—H5C2	108.1
C1A—C8A—C9A	110.8 (3)	C6C—C5C—H5C2	108.1
C1A—C8A—C7A	114.4 (3)	H5C1—C5C—H5C2	107.3
C9A—C8A—C7A	113.0 (3)	C7C—C6C—C5C	118.7 (13)
C1A—C8A—H8A	106.0	C7C—C6C—H6C1	107.6
C9A—C8A—H8A	106.0	C5C—C6C—H6C1	107.6
C7A—C8A—H8A	106.0	C7C—C6C—H6C2	107.6
O1A—C9A—C10A	108.1 (3)	C5C—C6C—H6C2	107.6
O1A—C9A—C8A	107.0 (3)	H6C1—C6C—H6C2	107.1
C10A—C9A—C8A	114.7 (3)	C7C—C14C—H14G	109.5
O1A—C9A—H9A	109.0	C7C—C14C—H14H	109.5
C10A—C9A—H9A	109.0	H14G—C14C—H14H	109.5
C8A—C9A—H9A	109.0	C7C—C14C—H14I	109.5
C16A—C10A—C11A	123.0 (4)	H14G—C14C—H14I	109.5
C16A—C10A—C9A	121.5 (4)	H14H—C14C—H14I	109.5
C11A—C10A—C9A	115.4 (3)	C7C—C15C—H15G	109.5
C10A—C11A—C12A	110.8 (3)	C7C—C15C—H15H	109.5
C10A—C11A—H11A	109.5	H15G—C15C—H15H	109.5
C12A—C11A—H11A	109.5	C7C—C15C—H15I	109.5
C10A—C11A—H11B	109.5	H15G—C15C—H15I	109.5
C12A—C11A—H11B	109.5	H15H—C15C—H15I	109.5
H11A—C11A—H11B	108.1	C14C—C7C—C15C	107.7 (19)
C1A—C12A—C11A	110.4 (3)	C14C—C7C—C6C	108.4 (15)
C1A—C12A—H12A	109.6	C15C—C7C—C6C	109.3 (13)
C11A—C12A—H12A	109.6	C14C—C7C—C8B	103.9 (15)
C1A—C12A—H12B	109.6	C15C—C7C—C8B	117.9 (14)
C11A—C12A—H12B	109.6	C6C—C7C—C8B	109.3 (15)
H12A—C12A—H12B	108.1	C1B—C8B—C9B	110.1 (3)
C3A—C13A—H13A	109.5	C1B—C8B—C7C	113.0 (8)
C3A—C13A—H13B	109.5	C9B—C8B—C7C	109.1 (6)
H13A—C13A—H13B	109.5	C1B—C8B—C7B	115.2 (8)
C3A—C13A—H13C	109.5	C9B—C8B—C7B	118.3 (7)
H13A—C13A—H13C	109.5	C1B—C8B—H8B	103.7
H13B—C13A—H13C	109.5	C9B—C8B—H8B	103.7
C7A—C14A—H14A	109.5	C7B—C8B—H8B	103.7
C7A—C14A—H14B	109.5	O1B—C9B—C10B	105.5 (4)
H14A—C14A—H14B	109.5	O1B—C9B—C8B	109.2 (3)
C7A—C14A—H14C	109.5	C10B—C9B—C8B	114.2 (3)
H14A—C14A—H14C	109.5	O1B—C9B—H9B	109.3
H14B—C14A—H14C	109.5	C10B—C9B—H9B	109.3
C7A—C15A—H15A	109.5	C8B—C9B—H9B	109.3
C7A—C15A—H15B	109.5	C16B—C10B—C11B	123.6 (7)
H15A—C15A—H15B	109.5	C16B—C10B—C9B	121.1 (7)
C7A—C15A—H15C	109.5	C11B—C10B—C9B	115.2 (4)
H15A—C15A—H15C	109.5	C10B—C11B—C12B	110.7 (4)
H15B—C15A—H15C	109.5	C10B—C11B—H11C	109.5
C10A—C16A—H16A	120.0	C12B—C11B—H11C	109.5
C10A—C16A—H16B	120.0	C10B—C11B—H11D	109.5
H16A—C16A—H16B	120.0	C12B—C11B—H11D	109.5
C9B—O1B—H1B	109.5	H11C—C11B—H11D	108.1
C2B—C1B—C12B	115.5 (3)	C11B—C12B—C1B	110.5 (3)
C2B—C1B—C8B	119.8 (3)	C11B—C12B—H12C	109.6
C12B—C1B—C8B	114.3 (3)	C1B—C12B—H12C	109.6
C2B—C1B—C3B	58.9 (3)	C11B—C12B—H12D	109.6
C12B—C1B—C3B	119.3 (3)	C1B—C12B—H12D	109.6
C8B—C1B—C3B	118.2 (3)	H12C—C12B—H12D	108.1
C3B—C2B—C1B	61.0 (2)	C3B—C13B—H13D	109.5
C3B—C2B—Cl1B	120.7 (3)	C3B—C13B—H13E	109.5
C1B—C2B—Cl1B	122.5 (3)	H13D—C13B—H13E	109.5
C3B—C2B—Cl2B	119.4 (3)	C3B—C13B—H13F	109.5
C1B—C2B—Cl2B	120.0 (3)	H13D—C13B—H13F	109.5
Cl1B—C2B—Cl2B	107.4 (2)	H13E—C13B—H13F	109.5
C2B—C3B—C4B	118.2 (4)	C10B—C16B—H16C	120.0
C2B—C3B—C13B	119.2 (4)	C10B—C16B—H16D	120.0
C4B—C3B—C13B	112.3 (3)	H16C—C16B—H16D	120.0
C2B—C3B—C1B	60.1 (2)		

### Hydrogen-bond geometry (Å, º)

**Table:**

*D*—H···*A*	*D*—H	H···*A*	*D*···*A*	*D*—H···*A*
O1*A*—H1*A*···O1*B*^i^	0.82	1.98	2.791 (4)	169
C8*A*—H8*A*···Cl1*A*	0.98	2.68	3.228 (4)	116
O1*B*—H1*B*···O1*A*	0.82	2.04	2.835 (4)	162
C8*B*—H8*B*···Cl1*A*	0.98	2.78	3.691 (4)	156
C8*B*—H8*B*···Cl1*B*	0.98	2.63	3.238 (4)	120
O1*A*—H1*A*···O1*B*^i^	0.82	1.98	2.791 (4)	169
C8*A*—H8*A*···Cl1*A*	0.98	2.68	3.228 (4)	116
O1*B*—H1*B*···O1*A*	0.82	2.04	2.835 (4)	162
C8*B*—H8*B*···Cl1*A*	0.98	2.78	3.691 (4)	156
C8*B*—H8*B*···Cl1*B*	0.98	2.63	3.238 (4)	120

Symmetry code: (i) −*x*+1, −*y*−1, *z*.
